# Effects of Myostatin Mutation on Onset of Laying, Egg Production, Fertility, and Hatchability

**DOI:** 10.3390/ani11071935

**Published:** 2021-06-29

**Authors:** Joonbum Lee, Dong-Hwan Kim, Andrew M. Brower, Izzy Schlachter, Kichoon Lee

**Affiliations:** 1 Department of Animal Sciences, The Ohio State University, Columbus, OH 43210, USA; lee.3920@osu.edu (J.L.); kim.4094@osu.edu (D.-H.K.); brower.55@buckeyemail.osu.edu (A.M.B.); schlachter.17@buckeyemail.osu.edu (I.S.); 2 The Ohio State University Interdisciplinary Human Nutrition Program, The Ohio State University, Columbus, OH 43210, USA

**Keywords:** myostatin, quail, egg production, layer

## Abstract

**Simple Summary:**

Poultry can be classified as broilers for meat production and layers for egg production. Modern poultry farming improved economically important traits of broilers and layers by breeding and genetic selection. Myostatin (MSTN) has gained attention as a potential selection marker for higher meat production in the poultry industry, because MSTN mutant chickens and quail showed increased muscle mass. In this study, the effect of MSTN mutation on egg production was investigated to evaluate potential use of MSTN for higher egg production in the layer industry. MSTN homozygous mutant quail showed a significantly delayed onset of egg laying, a higher egg weight, and a lower number of eggs produced during the active laying period compared to wild-type quail. However, there were no significant differences in total egg production for 20 days, percentage proportion of egg white and yolk in egg weight, and egg fertility, and hatchability between MSTN mutant and WT quail. Although a clear benefit on egg production by MSTN mutation in quail was not revealed, this study provided useful information to understand the productive performance of MSTN mutant hens.

**Abstract:**

Increased body weight and muscle mass, along with improved feed efficiency, by myostatin (MSTN) mutation in quail, supports the potential use of MSTN as a selection marker for higher meat yield in the poultry industry. Although economically important traits of broilers have been studied using recently generated MSTN mutant quail, the effect of MSTN mutation on egg production has not yet been investigated. In this study, several economically important traits of layers, including egg production, reproduction, and body composition of hens, were compared between MSTN homozygous mutant, heterozygous mutant, and wild-type (WT) quail. In terms of egg production, MSTN homozygous mutant quail, showing significantly delayed onset of egg laying, laid significantly heavier eggs, but a significantly lower number of eggs compared to WT quail for 20 days after 3 months of age, resulting in similar total egg production among groups. In addition, the percentage proportion of egg white and yolk in egg weight were similar among groups. Furthermore, similar fertility and hatchability of eggs from MSTN homozygous mutant breeding pairs and WT breeding pairs indicated normal reproductive function of MSTN mutant quail. These findings will provide scientific rationales for the consideration of MSTN as a potential selection marker for layers in the poultry industry.

## 1. Introduction

Since the discovery of the anti-myogenic function of myostatin (MSTN) in mammals, MSTN was considered as a potential selection marker for a higher meat yield in the livestock industry. Unlike other MSTN mutant mammals reported and studied for a relatively long time [1,2,3], genome-editing of the MSTN gene in avian species including quail and chickens has been recently generated and studied mainly regarding growth rate and development of muscle and adipose tissue [4,5]. Therefore, other economically important traits of poultry need to be further investigated to consider the usage of MSTN as a potential selection marker in the poultry industry. In addition to increased body and muscle weight, improved feed efficiency in MSTN homozygous mutant quail was also reported in our recent study [6]. Although previous studies demonstrated positive effects of MSTN mutation on economically important traits of broilers, the effect of MSTN mutation on egg production traits of layers has not been reported. 

In addition to the meat production of broilers, commercial egg production of layers is an important sector in the poultry industry. Therefore, an increase in egg production has been achieved by improving layer genetics and a farm management system [7]. In modern commercial poultry farming, optimal management systems have been practiced to control factors affecting egg production such as environmental factors of poultry farms and physical characteristics of egg laying hens. Especially, feed is one of the main concerns in poultry farming because not only does feed comprise a major portion of the total production cost in the poultry industry, but also body weight and fatness affect the onset of egg production [8]. Body weight has been known to affect egg size positively and fat accumulation is increased prior to egg laying [9,10]. However, Overweight with excessive fat accumulation could negatively affect egg laying performance [11]. In our previous study, body and fat weight of MSTN homozygous mutant females were approximately 15% heavier and 30% lower than wild-type (WT) females, respectively, at 6 weeks old, when egg laying is about to be initiated in quail [4]. Because MSTN mutation affects both body weight and fatness of hens, the effect of MSTN mutation on egg production of MSTN homozygous mutant quail needs to be investigated.

In addition to the productive traits, functional traits of layers, such as fertility and hatchability of eggs, are also important for higher profitability in poultry industries. Although the direct effect of MSTN mutation on reproductive performance in birds has not been reported, fertility and hatchability were significantly higher in the small egg size group compared to the large egg size group in broiler breeder chickens [12]. Likewise, the egg weight of MSTN mutant quail and resultant fertility and hatchability can be affected by MSTN mutation. These unknown effects of MSTN mutation on reproductive traits of avian species can now be studied using recently generated MSTN mutant quail. Therefore, the aim of this study was to investigate the effect of MSTN mutation on egg production traits of female quail by examination of age of first egg, egg weight, total number of eggs produced during the active laying period, weights of egg white and yolk, and egg fertility and hatchability.

## 2. Materials and Methods

### 2.1. Animal Care

All animal care protocol and experimental procedures were approved by the Institutional Animal Care and Use Committee at The Ohio State University (Protocol 2019A00000024). Japanese quail (*Coturnix japonica*) with MSTN mutation were generated from our previous study [4] and maintained at The Ohio State University Poultry Facility in Columbus. After raising quail as groups in brooder cages, 5 weeks old quail were transferred to small individual cages (12″ × 16″ × 16″) to record first egg laying dates and collect eggs from each quail. All quail were fed the same standard chicken diet produced by The Ohio State University’s Research Feed Mill in Wooster and euthanized via CO_2_ inhalation. 

### 2.2. Egg Collection and Tissue Sampling

After the initiation of laying approximately at 6 weeks old, quail were raised until 3 months of age to give enough time for stabilization of egg weight and number during the active laying period. Then, eggs were collected daily for 20 days from 10 homozygous and 9 heterozygous mutants, and 9 WT female quail at 3 months old to count the numbers of eggs and measure the weights of whole eggs, egg whites, and egg yolks. Total egg amount was calculated by multiplying average egg weights and average number of eggs produced for 20 days. After collection of eggs, pectoralis major, leg fat, and abdominal fat were sampled from the same quail at 4 months old. To analyze the onset of egg laying, additional female quail were raised and dates of first egg laying were recorded from 25 MSTN homozygous and 40 heterozygous mutants, and 22 WT female quail.

### 2.3. Egg Fertility 

Another batch of MSTN homozygous mutant and WT quail groups were prepared. At 3 months old, 9 pairs of MSTN homozygous mutant male and female quail and 7 pairs of WT male and female were mated in separate small cages, respectively. Then, eggs were collected daily from each breeding pair and incubated weekly. Egg fertility and hatchability were measured by counting total incubated eggs, hatched quail, and developed embryos from unhatched eggs.

### 2.4. Statistical Analyses

All data were expressed as means ± SEM. The Student *t*-test was used for statistical analysis to compare fertility and hatchability of eggs. One-way ANOVA followed by Tukey’s multiple comparisons test were used for comparisons of onset of egg laying, egg weights, numbers, and egg white and yolk among homozygous and heterozygous mutants, and WT groups. All statistical analyses were performed by the GraphPad PRISM (version. 6.02, Graphpad Software, La Jolla, CA, USA) and detailed numbers of samples were described in each of the Figure Legends. 

## 3. Results

### 3.1. Egg Production Traits of MSTN Mutant Quail

**Figure 1:**
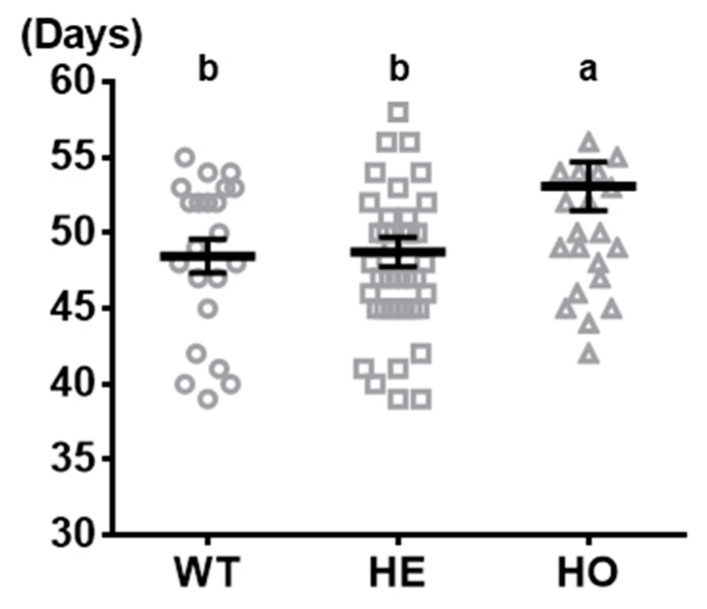
Comparison of first egg laying dates of wild-type (WT), MSTN heterozygous mutant quail (HE) and MSTN homozygous mutant quail (HO). Values present means ± SEM. *n* = 22 WT females, 40 HE females, and 25 HO females. One-way ANOVA was used for statistical analysis by the GraphPad PRISM. ^a,b^ Means that have no superscript in common in a graph are different (*p* < 0.05).

Average ages of hens reaching onset of first egg laying were very similar between WT (48.45 days) and MSTN heterozygous mutant hens (48.73 days). However, MSTN homozygous mutant quail had approximately 5 days of significant delay in onset of egg laying (53.08 days) compared to heterozygous mutant and WT quail (Figure 1). 

**Figure 2:**
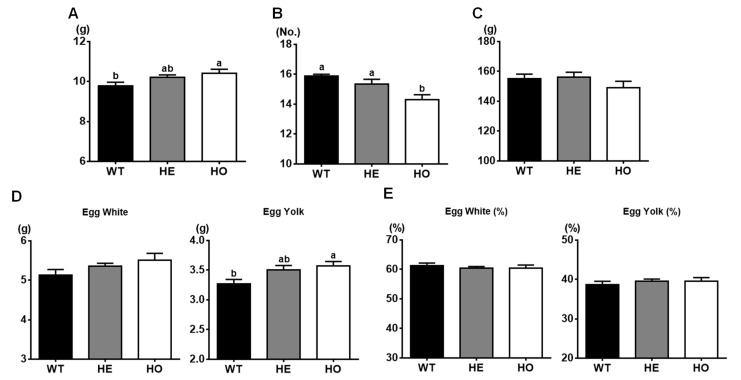
Comparisons of egg quality and quantity traits of WT, HE, and HO female quail from 90 to 110 days of age. (**A**) Egg weights. (**B**) Egg numbers. (**C**) Total egg production. (**D**) Weights of egg whites and yolks. (**E**) Percentage proportions of egg whites and yolks in egg weights. Values present means ± SEM. *n* = 9 WT females, 9 HE females, and 10 HO females. One-way ANOVA was used for statistical analysis by the GraphPad PRISM. ^a,b^ Means that have no superscript in common in a graph are different (*p* < 0.05). No.: Number of eggs.

In terms of egg weight, homozygous mutant quail laid significantly heavier (7%) eggs compared to WT quail, and the weights of eggs from heterozygous mutant quail were intermediate (Figure 2A). On the contrary, total number of eggs produced for 20 days during the active laying period were significantly lower in homozygous mutant quail compared to heterozygous mutant and WT quail (Figure 2B), resulted in similar total amount of eggs among groups (Figure 2C). Although the egg yolk weights of homozygous mutant quail were significantly heavier than the one from WT quail (Figure 2D), the percentage proportion of egg white and yolk in egg weight were similar among groups (Figure 2E), showing an increase of egg white and yolk weights in proportion to the egg weight of different genotypes. 

### 3.2. Fat and Muscle of MSTN Mutant Quail

Body and pectoralis major muscle weights of MSTN homozygous mutant quail were significantly heavier than those of heterozygous mutant and WT quail at 4 months old (Table 1). On the contrary, leg and abdominal fat weights and percentages of homozygous mutant quail were significantly lower than those of WT quail. However, leg and abdominal fat weights and percentages of MSTN heterozygous mutant quail were intermediate between those of homozygous mutant and WT quail.

### 3.3. Fertility and Hatchability of Eggs from MSTN Mutant Quail

There was no significant difference in fertility and hatchability of eggs from MSTN homozygous mutant breeding pairs and WT breeding pairs (Table 2). 

## 4. Discussion

The positive correlation between egg weight and body weight of hens has been reported in chickens and turkeys [9,13]. Furthermore, the quail line selected for increased body weight laid heavier eggs compared to quail line with selection for decreased body weight [14]. Likewise, the average egg weight during the active laying period of MSTN homozygous mutant quail was significantly greater than that of WT quail (Figure 2A) and the body weight of homozygous mutant females was approximately 15% and 10% higher than that of WT females at 6 weeks old [4], right before the onset of egg laying, and at 4 months old, active laying period, respectively.

On the contrary, total number of eggs produced for 20 days during the active laying period were significantly lower in MSTN homozygous mutant quail compared to WT quail (Figure 2B). Similarly, egg production of the quail line selected for higher body weight was decreased [14]. However, similar total egg production (Figure 2C), with similar percentage proportion of egg white and yolk (Figure 2E), for 20 days among groups might indicate the capacity of egg production might not be affected by MSTN mutation, but it took more time to produce bigger eggs from heavier quail.

Similar percentage proportions of egg white and yolk among groups indicated that the capacity of egg white and yolk production was not compromised by MSTN mutation. Notably, the egg yolk weight of MSTN homozygous mutant eggs were significantly greater than those of WT eggs (Figure 2D). Indeed, positive correlation between body weight and yolk weight were also shown in the quail line selected for higher body weight [14]. This can be partially explained by more body fat of the quail line selected for higher body weight compared to the quail line selected for lower body weight [15], because moderate correlation between the body fat contents and egg yolk percentage were observed in white and brown egg layer chickens [10]. However, MSTN homozygous mutant quail had significantly lower leg and abdominal fat compared to those of WT quail (Table 1), indicating lipid mobilization for yolk formation was not compromised by lower fat accumulation from MSTN homozygous mutation. Although lower leg and abdominal fats of MSTN homozygous mutant quail did not negatively affect the egg production during the active laying period, significantly delayed onset of egg laying of MSTN homozygous mutant quail compared to WT quail might be associated with low fat accumulation, because negative correlation between body fat content and onset of egg laying were observed in broiler breeder chickens [8]. Furthermore, increased fat accumulation during the onset of the first egg laying period have been reported in quail [16]. It has been generally accepted that there are multiple threshold traits, including age, body weight, and body composition, needs to be reached for the onset of egg laying in quail and chickens [17,18]. According to the delayed egg laying of MSTN homozygous quail in our study, however, body composition changed by weight gain, mainly body fat accumulation, would be a more critical factor for the onset of egg laying than the body weight itself. 

In broiler breeder male and female chickens, Overweight negatively affects their reproductive traits [11], and thus intensive management is required to prevent overconsumption of feed to attain optimal body weight for high reproductive performance. Although improved feed efficiency and low leg and abdominal fats of MSTN homozygous mutant female indicated increased body weight is not caused by overconsumption of feed [4,6], the effect of MSTN mutation on egg fertility and hatchability was examined in this study. There was no significant difference on egg fertility and hatchability among groups, preventing potential problems of reproductive traits on breeder line with MSTN mutation. In mammals, double-muscled cows with MSTN mutation have increased risk of dystocia due to heavier weight of their offspring, but the quality of semen has not been affected by MSTN mutant in pigs. Therefore, fetal size of MSTN mutant animals can influence the reproductive traits of MSTN animals indirectly. However, birds lay eggs and the size is determined by the capacity of reproductive organs and, thus the indirect effect of egg size on the reproductive traits of MSTN birds can be negligible in avian species. In addition, when chicken lines are selected for abdominal fat content, the lean chicken male and female showed better reproductive traits with higher fertility and hatchability [19]. Likewise, low fat accumulation by MSTN homozygous mutation might contribute to the normal fertility and hatchability in MSTN homozygous mutant quail. 

## 5. Conclusions

In this study, we investigated the effect of MSTN mutation on egg production traits to provide information about potential usage of MSTN as a selective marker in the layer industry. Although MSTN homozygous mutant quail showed significantly heavier eggs with normal fertility and hatchability compared to WT quail, there was a negative effect on the onset of egg laying and egg production during the active laying period in MSTN homozygous mutant quail. While the positive effects of MSTN mutation on egg production cannot be concluded in this study due to various implications, consideration of MSTN as a potential selection marker for layers will remain a decision of the layer breeding companies depending on their prior selection criteria. 

## Figures and Tables

**Table 1:** Adipose and muscle weights, and percentage of each tissue compared to body weight of WT, HE, and HO quail hens at 4 months old.

Items	WT	HE	HO
Body Weight (BW, g)	131.02 ± 1.90 ^b^	131.82 ± 1.57 ^b^	145.09 ± 2.12 ^a^
Leg Fat (g)	0.44 ± 0.06 ^a^	0.35 ± 0.04 ^ab^	0.28 ± 0.03 ^b^
Leg Fat Percentage (%)	0.36 ± 0.04 ^a^	0.27 ± 0.03 ^ab^	0.19 ± 0.02 ^b^
Abdominal Fat (g)	0.22 ± 0.03 ^a^	0.19 ± 0.02 ^ab^	0.14 ± 0.02 ^b^
Abdominal Fat Percentage (%)	0.17 ± 0.02 ^a^	0.15 ± 0.02 ^ab^	0.10 ± 0.01 ^b^
Pectoralis Major (PM, g)	17.34 ± 0.43 ^b^	17.93 ± 0.34 ^b^	22.21 ± 0.52 ^a^
PM Percentage (%)	13.23 ± 0.24 ^b^	13.60 ± 0.20 ^b^	15.30 ± 0.21 ^a^

The values are average ± SEM. *n* = 9 WT, 9 HE and 10 HO. One-way ANOVA was used for statistical analysis by the GraphPad PRISM. ^a,b^ Means that have no superscript in common in a row are different (*p* < 0.05).

**Table 2:** Comparisons of fertility and hatchability of eggs from WT and HO breeding pairs.

Items	WT x WT	HO x HO
Breeding pairs	7	9
Total incubated egg/pair	27.43 ± 1.56	27 ± 0.97
Fertility (%)	87.03 ± 3.46	85.47 ± 4.95
Hatchability (%)	67.12 ± 4.66	67.95 ± 4.96

The values are average ± SEM. Student *t*-test was used for statistical analysis by the GraphPad PRISM (*p* < 0.05).

## Data Availability

Data is contained within the article.
